# Shear bond strength and debonding characteristics of metal and ceramic brackets bonded with conventional acid-etch and self-etch primer systems: An *in-vivo* study

**DOI:** 10.4317/jced.52658

**Published:** 2016-02-01

**Authors:** Behnam Mirzakouchaki, Sajjad Shirazi, Reza Sharghi, Samaneh Shirazi, Mahsan Moghimi, Shirin Shahrbaf

**Affiliations:** 1 Associate Professor, Department of Orthodontics, Faculty of Dentistry, Tabriz University of Medical Sciences, Tabriz, Iran; 2 Lecturer and Faculty Member, Faculty of Dentistry, Tabriz University of Medical Sciences, Tabriz, Iran; 3 Research Fellow, Dental and Periodontal Research Center, Tabriz University of Medical Sciences, Tabriz, Iran; 4 Assistant professor of orthodontics, Dental Caries Prevention Research Center, Qazvin University of Medical Sciences, Qazvin, Iran; 5 Research Assistant, Faculty of Paramedicine, Tabriz University of Medical Sciences, Tabriz, Iran; 6 Post Graduate Student, Department of Orthodontics, Faculty of Dentistry, Tabriz University of Medical Sciences, Tabriz, Iran; 7 Clinical Teacher, Academic Unit of Restorative Dentistry, Sheffield Dental School, Sheffield, UK

## Abstract

**Background:**

Different *in-vitro* studies have reported various results regarding shear bond strength (SBS) of orthodontic brackets when SEP technique is compared to conventional system. This *in-vivo* study was designed to compare the effect of conventional acid-etching and self-etching primer adhesive (SEP) systems on SBS and debonding characteristics of metal and ceramic orthodontic brackets.

**Material and Methods:**

120 intact first maxillary and mandibular premolars of 30 orthodontic patients were selected and bonded with metal and ceramic brackets using conventional acid-etch or self-etch primer system. The bonded brackets were incorporated into the wire during the study period to simulate the real orthodontic treatment condition. The teeth were extracted and debonded after 30 days. The SBS, debonding characteristics and adhesive remnant indices (ARI) were determined in all groups.

**Results:**

The mean SBS of metal brackets was 10.63±1.42 MPa in conventional and 9.38±1.53 MPa in SEP system, (*P*=0.004). No statistically significant difference was noted between conventional and SEP systems in ceramic brackets. The frequency of 1, 2 and 3 ARI scores and debonding within the adhesive were the most common among all groups. No statistically significant difference was observed regarding ARI or failure mode of debonded specimens in different brackets or bonding systems.

**Conclusions:**

The SBS of metal brackets bonded using conventional system was significantly higher than SEP system, although the SBS of SEP system was clinically acceptable. No significant difference was found between conventional and SEP systems used with ceramic brackets. Total SBS of metal brackets was significantly higher than ceramic brackets. Due to adequate SBS of SEP system in bonding the metal brackets, it can be used as an alternative for conventional system.

** Key words:**Shear bond strength, Orthodontic brackets, Adhesive remnant index, self-etch.

## Introduction

Direct bonding of orthodontic brackets can be achieved by the micromechanical adhesion of a resin-based material to etched enamel (1). Bonding materials should penetrate into the enamel porosities and have simple manipulation and dimensional stability. They should maintain adequate bond strength to prevent bonding failure and subsequent treatment cessation while withstanding masticatory forces, arch-wire induced stresses and forces induced by incorrect use of the appliance by patient (1). Furthermore, the bond strength should not be so much high to prevent bracket debonding and damage the tooth structure at the end of orthodontic treatment (2).

Damage to the enamel surface when debonding orthodontic brackets has been a clinical concern. Ideally, bond failure at the bracket-adhesive interface should occur without damaging the enamel surface. Bracket debonding takes place in regions closer to the enamel-adhesive interface with the increased bond strength. Furthermore, increased forces during debonding cause more stresses and cracks in the enamel surface (3).

During the bonding of orthodontic brackets to enamel, conventional adhesive systems use three different agents: an enamel conditioner, a primer solution and an adhesive resin (4). In the other hand, the self-etching primer (SEP) systems have been introduced, which combine acid and primer and simplify the bonding procedure, reduce chair time and technique-sensitivity, risk of saliva contamination and side-effects of acid etching while maintain similar rates of etching depth and primer penetration (1,5). Furthermore, the major role of the operator in the conventional technique on the bracket’s shear bond strength (SBS) has been eliminated with the introduction of SEP technique (6).

Different results have been reported regarding SBS of orthodontic brackets when SEP technique is compared to conventional system. Bishara *et al.* (5) and Buyukyilmaz *et al.* (7) evaluated the effect of conventional and SEP systems on the SBS of orthodontic brackets and reported higher values of SBS using SEP system. Other studies concluded similar rates of SBS for both conventional and SEP techniques (8-10). Aljubouri *et al.* (11) and Korbmacher *et al.* (12) reported that the SEP system had a lower SBS than the conventional system when bonding metal brackets. Despite the reported differences, the acquired values of bond strength between adhesive and enamel were clinically acceptable according to these studies (4,12).

Most of studies concerning orthodontic brackets SBS are *in-vitro*, using extracted teeth due to the difficulties of the assessing volunteer patients or longer study periods in the oral cavity. The generalization of *in-vitro* results for oral condition is limited for the problems associated with complete isolation, moist oral cavity, attrition induced by the foods, bacterial activity of oral cavity and suitable insertion of brackets on the tooth surfaces.

In this *in-vivo* study we aimed to simulate the real treatment conditions. The purposes of this study were: 1- To compare the SBS of ceramic and metal brackets bonded with SEP and conventional systems. 2- To determine debonding characteristics and mode of bond failure in brackets bonded with the above systems.

## Material and Methods

The study procedure was explained to the patients. They voluntarily participated in this study and signed an informed consent. The approval for the study was also obtained from the research ethics committee of the university (Reference number: 12130428).

Considering α = 0.05 and power = 80 per cent and 0.6 as maximum tolerable error rate (d) lead to a required sample size of 25 for each group, which was increased to 30 to improve the validity of the study and compensate for probable undesirable debonding.

One hundred and twenty teeth comprising 60 maxillary and 60 mandibular first premolars, in 30 orthodontic patients aged 18-29 years old (17 females and 13 males) were included in this study. The criteria for selection was intact and sound buccal enamel; with no caries, attrition, crack, restoration, congenital anomalies and structural defects as shown by trans-illumination examinations, and no previous treatment with chemical agents. The teeth with noticeable differences in shape and size were excluded. Moreover, the cases with inadvertent brackets debonding before extraction as well as the teeth showing occlusal contact on the brackets were excluded too.

The subjects were randomly allocated to study groups, then metal and ceramic brackets (3M Unitek, Monrovia, USA) were bonded randomly either with conventional or SEP system (3M Unitek, Monrovia, USA) on the four selected teeth. This was to reduce the effect of interfering factors such as occlusal forces and bracket positioning and for randomly distribution of various bracket and bonding methods in all quadrants.

There were two experimental (SEP) and two control (conventional acid-etching) groups, including 120 samples. Each group consisted of 30 teeth as follows: 1- Metal brackets bonded with conventional system. 2- Metal brackets bonded with SEP system. 3- Ceramic brackets bonded with conventional system. 4- Ceramic brackets bonded with SEP system.

Premolar metal brackets with mechanical retention and the base surface area of 12 mm2 and premolar ceramic brackets with mechanical retention and base surface area of 14.6 mm2 were bonded to the studied teeth using one of the methods.

The teeth surfaces were polished with pumice, thoroughly washed and dried completely then isolated with cotton roll and suction. In the experimental groups, the SEP was placed on the enamel according to the manufacturer’s instruction by means of special brush followed by drying for two seconds using gentle air flow after 15 seconds. The enamel surface was kept wet during the procedure to facilitate the monomer penetration. The adhesive paste (3M Unitek, Monrovia, USA) was applied to bracket base, which was seated by the application of moderate compressive force for 10 seconds in order to obtain smooth steady adhesive thickness on the enamel surface. In the control groups, the teeth were etched with 37 per cent phosphoric acid (3M Unitek, Monrovia, USA) according to manufacturer’s instruction then were thoroughly washed by water spray for 15 seconds. The excess water was removed by gentle air flow from 2 cm distance for 10 seconds. When the white chalky surface of enamel was observed, the primer solution (3M Unitek, Monrovia, USA) was applied on the teeth. The brackets were then seated as in the first group using the same adhesive paste.

The brackets were cured with halogen light curing device (Ivoclar Vivadent, Amherst, USA). The light was applied for 10 seconds at mesial, distal, occlusal and gingival aspects of the brackets.

The bonded brackets were incorporated into the wire (0.016 inch round NiTi) during the study period to simulate the real orthodontic treatment condition. All teeth were maintained in the patients’ mouth for 30 days prior extraction and were extracted using surgical elevators (Aesculap, Tuttlingen, Germany) to prevent contact with the brackets and debonding. The extracted teeth were stored in 0.1% thymol solution (Thymol Mylan, Seiyaku, Japan) to prevent bacterial growth and dehydration until they were embedded in a self-cure acrylic (Ivoclar Vivadent, Naturno BZ, Italy) block up to cementoenamel junction in a way that the bracket base vertical axis, in the contact point of the base to the teeth, vertically crossed the horizontal line. They were then coded from 1 to 120.

The blocks were placed in the Hounsfield Test Equipment (HTE, Surrey, England) and fixed in lower grip of the machine. A steel rod with the cutting edge of 0.5 mm was attached to the crosshead of the machine. Each tooth labial surface was oriented to be parallel to the force during the SBS test. The tooth placement in the machine was examined by two operators. An occlusogingival load was applied to the bracket, producing a shear force at the bracket-tooth interface. The force was measured in Newtons at a crosshead speed of 0.5 mm/min and divided by the surface area of the brackets pad to calculate the SBS in megapascals (MPa).

Debonding areas in the bracket surface and enamel were assessed using a light stereomicroscope (Olympus,Tokyo, Japan) under a 40x magnification to determine debonding characteristics and the amount of remaining adhesive using adhesive remnant index (ARI). ARI included five scores as follows: 1, all adhesive was remained on the enamel surface and the bracket had no remaining adhesive. 2, more than 90 per cent of adhesive was remained on the enamel surface. 3, less than 90 per cent and more than 10 per cent of adhesive was remained on the enamel surface. 4, less than 10 per cent of adhesive was remained on the enamel surface. 5, no adhesive was remained on the enamel surface.

The bond strength values were examined for normality using Kolmogorov-Smirnov analysis. ANOVA and Student-t tests were used to analyze the bond strength differences among study groups. Mann-Whitney U test was used to assess the differences in the ARI scores between groups. Failure mode of debonded specimens among study groups was analyzed using Chi-square test. A *P*-value<0.05 was selected as the level of statistical significance and the data analysis was performed using SPSS version 16 (IBM, Chicago, USA).

## Results

112 teeth in 28 patients (22.85±2.88 years old, 15 females and 13 males) were available for evaluation. One patient (4 teeth) did not attend for extraction. Two brackets were debonded during the study and two failed during extraction.

Bracket type and bonding system had a significant effect on SBS (*P*=0.0001 and *P*=0.005 respectively). The mean SBS of metal and ceramic brackets in the two systems was 9.99±1.59 MPa and 7.07±1.18 MPa respectively.

**Figure 1:**
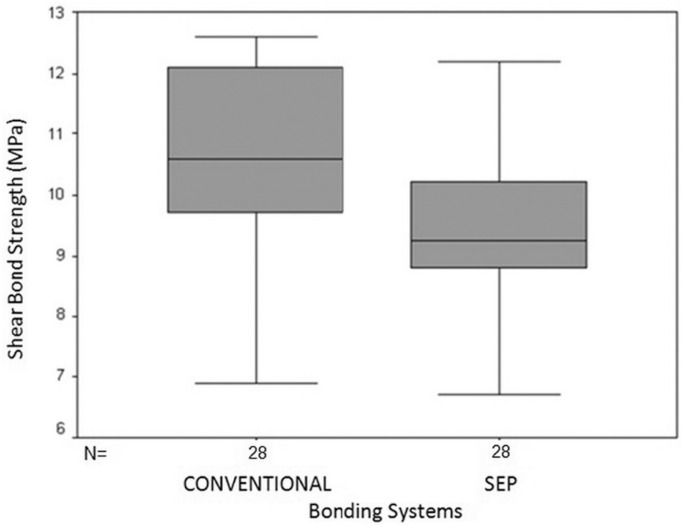
Mean and 95% confidence interval of SBS of metal brackets using two bonding systems.

**Figure 2:**
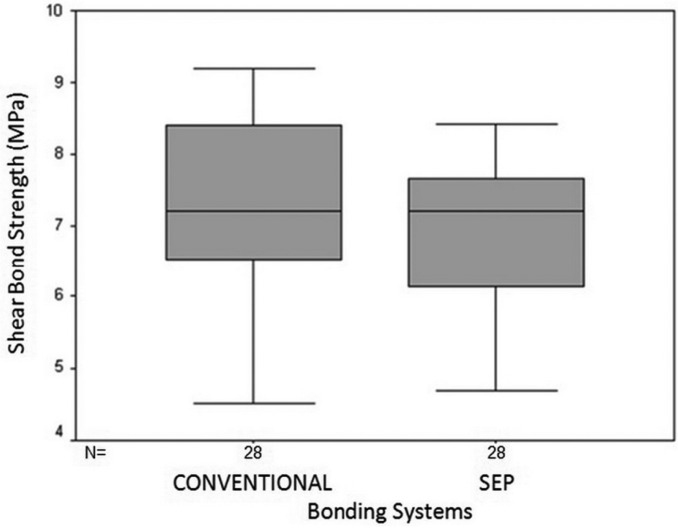
Mean and 95% confidence interval of SBS of ceramic brackets using two bonding systems.

There was significant difference between conventional and SEP systems when metal brackets were used (*P*=0.004). In these brackets, the mean SBS of specimens bonded with conventional system was 10.63±1.42 MPa vs. 9.38±1.53 MPa in teeth bonded with SEP technique. However, no significant difference was found between conventional and SEP systems in ceramic brackets (*P*>0.05). In these brackets, the mean SBS was 7.2±1.32 MPa in the conventional and 6.92±1.03 in the SEP system (Figs. 1,2).

In metal brackets, ARI scores of 1 and 5 showed the most and the least frequencies respectively (37.5% and 1.8%). In ceramic brackets, 60.8 per cent of specimens showed ARI scores of 1 or 2 and 3.5 per cent of which presented ARI score of 5. Regarding ARI scores, there was no significant difference (*P*>0.05) between specimens bonded with metal and ceramic brackets ( Table 1).

**Table 1:**
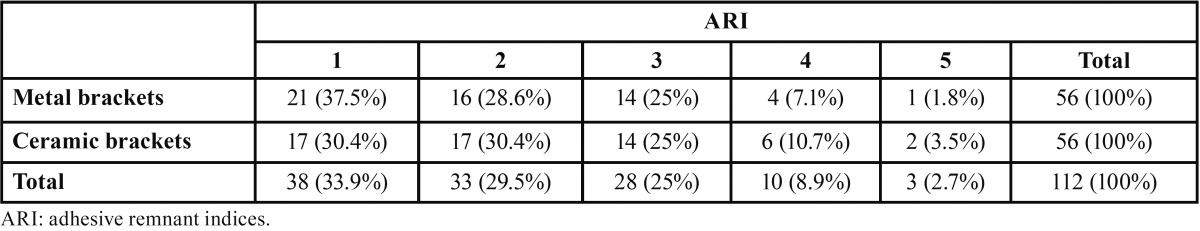
ARI score in the samples bonded with the metal and ceramic brackets.

ARI scores of 1 and 5 had the most and the least frequencies in the teeth bonded with conventional system respectively (32.2% and 3.5%). Similar results were observed in the teeth bonded by SEP technique with 35.7 per cent showing score 1 and 1.8 per cent score 5. No significant difference (*P*>0.05) was noted between the two bonding techniques regarding ARI scores ( Table 2).

**Table 2:**
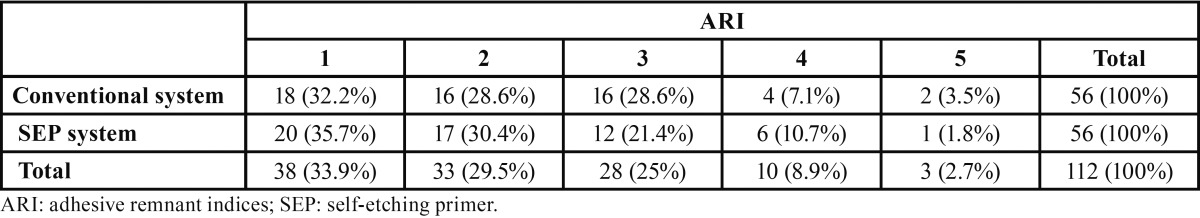
ARI score in the samples bonded with the conventional and SEP bonding systems.

The highest frequency of bonding failure was at the adhesive region and the least frequency at the enamel-adhesive interface. No significant difference (*P*>0.05) was observed between study groups ( Table 3, Table 4).

**Table 3:**
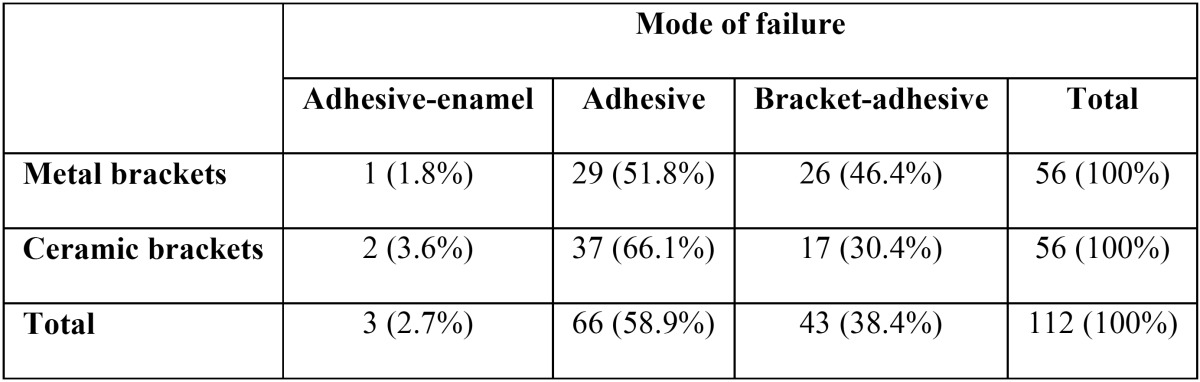
Failure mode of samples after shear bond strength test in metal and ceramic brackets.

**Table 4:**
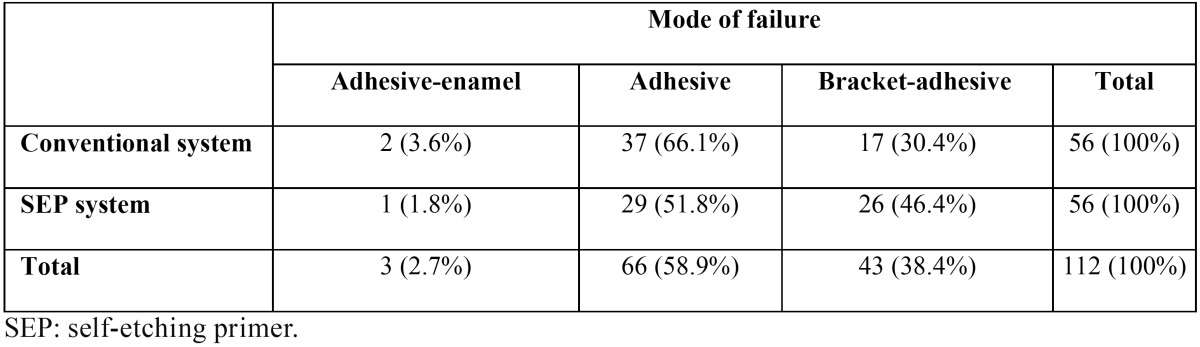
Failure mode of samples after shear bond strength test in conventional and SEP systems.

## Discussion

This *in-vivo* study was designed to compare the effect of conventional acid-etching and self-etching primer adhesive (SEP) systems on SBS and debonding characteristics of metal and ceramic orthodontic brackets. One of the most distinguishing features of this study was incorporation of bonded brackets into wire during study period which simulates the real oral condition.

This study showed no significant difference between conventional acid-etching and SEP systems regarding SBS of teeth bonded with ceramic brackets. However, slightly higher SBS values were noted in the conventional acid-etching technique compared to self-etching primer (7.2 MPa vs. 6.92 MPa). In contrast, mean SBS of metal brackets bonded with the SEP system was significantly lower (*P*=0.004) than conventional method (9.38 MPa vs. 10.63 MPa). Although there is no universally accepted minimum SBS for clinical orthodontic situations, some proposed SBS of 8-9 MPa to be adequate for orthodontic bracket bonding (13). As a result, our study indicated acceptable SBS in bonding of metal orthodontic brackets to enamel surface using self-etching primer but the bond strength achieved by ceramic brackets did not meet the criteria. Due to the higher values of bonding strength in metal brackets compared to ceramic ones, poor performance of SEP technique in ceramic brackets is related to bracket type rather than SEP technique. So, SEP system is capable to maintain adequate SBS compared to the conventional technique.

Cal-Neto *et al.* (14) reported no significant difference in the mean SBS of a conventional acid-etch system and a SEP technique. Some other studies demonstrated adequate SBS of self-etching primers (1,15,16). Mirzakouchaki *et al.* (9) reported lower SBS using self-etching primer for enamel preparation although it was acceptable for clinical conditions. Furthermore, Bishara *et al.* (4) found self-etching primer to have lower SBS, although it was adequate. However, others reported significantly lower bonding strength of SEP technique than conventional method (4,17,18). The differences may be attributed to the studied specimens (animal teeth, human teeth, anterior and posterior teeth), the assessed environment (oral cavity, laboratory condition), enamel surface preparation, the use of different adhesives, the time to calculate bond strength and debonding technique.

Adequate SBS of self-etching primer, as shown in the present study, can be related to simultaneous etching and priming of enamel surface in which primer penetrates into all depths of etching area, providing an excellent mechanical interlock. Self-etching primers simplify the clinical handling of adhesive systems by combining priming and etching. However, it can lead to significantly decreased bond strength of orthodontic brackets (4).

We found significantly higher SBS for metal brackets when compared to ceramic types. Ceramic brackets have been used to meet the patient’s aesthetic needs due to the tooth-colored structure, despite of decreased SBS compared to metal brackets. Mirzakouchaki *et al.* (9) reported significantly higher bonding strength for metal brackets in both SEP and conventional systems. However, Bishara and Olsen (19) and Kuang *et al.* (20) demonstrated no significant difference between bonding strength of two ceramic and metal brackets. Different regimen for load application, different bonding material, different adhesives and material preparation method and the use of thermocycling for specimen preparation are possibly responsible for variation of the results. It was shown that ceramic brackets of polycrystalline promote higher SBS than metal brackets while crystalline ceramic brackets produced the least SBS values (21). In spite of these findings, one study revealed no significant difference in the SBS of different types of ceramic brackets (22).

In our study the ARI scores in two techniques and bracket types had no significant difference. Furthermore, Failure mode of debonded specimens was similar in study groups with the failure at the adhesive region as the most frequent type in all groups. Cal-Neto *et al.* (14) reported no significant statistical difference regarding ARI scores between conventional and SEP techniques. However, Contradictory results had been reported regarding ARI scores following orthodontic bonding with the conventional acid phosphoric etching and SEP techniques (4,23,24). The amount of the remaining adhesive after orthodontic debonding is clinically important. With the occurrence of bond failure at the area closer to enamel and adhesive region or with the reduced amounts of remaining adhesive on the tooth surface, more stresses will occur at the enamel surface. ARI scores of 1, 2 and 3 were observed more frequently than 4 and 5 in this study. As no significant difference existed regarding ARI scores and failure mode of different groups, SEP system promoted similar results to the conventional method.

In the previous studies, *in-vitro* results of bond strength have been generalized to *in-vivo* conditions. However, the bond strength determined by the laboratory examinations may not be the exact indicator of the performance of different bonding systems in clinical situations. Some studies revealed significant difference between bond strength values achieved in laboratory and clinical conditions, suggesting *in-vitro* results must be interpreted with caution when used in clinical situations (25). Pickett, *et al.* showed that bond strength *in-vivo* were significantly less than those calculated *in-vitro* (26). Hajrassie and Khier reported lower bond strength in oral environment compared to *in-vitro* conditions (27). Possible reasons contributed to these variations are: the time of appliance use in the oral cavity, expose of the bonded brackets to acid and saliva, patients’ incorrect use of brackets and the role of masticatory forces, all of which influencing the bond strength values.

In overall, SEP technique offers decreased technique-sensitivity and clinical steps, improved handling of adhesive, decreased decalcification risk and white spot formation as well as adequate SBS. However, more *in-vivo* studies are required to assess the bond strength of SEP and conventional acid etching systems in ceramic and metal brackets with the matched conditions as possible. Longer periods of presence of brackets in oral cavity should be considered in future studies. Comparing results for pediatric and adult patients with the different bonding techniques should also be taken into account.

## Conclusions

The present *in-vivo* study showed that the use of SEP technique for bonding of metal orthodontic brackets may be considered as an alternative for conventional acid etching. However, SBS of ceramic brackets bonded with conventional and SEP systems was lower than the acceptable range.
